# Home Learning Environments of Children in Mexico in Relation to Socioeconomic Status

**DOI:** 10.3389/fpsyg.2021.626159

**Published:** 2021-03-19

**Authors:** María Inés Susperreguy, Carolina Jiménez Lira, Chang Xu, Jo-Anne LeFevre, Humberto Blanco Vega, Elia Verónica Benavides Pando, Martha Ornelas Contreras

**Affiliations:** ^1^Faculty of Education, Pontificia Universidad Católica de Chile, Santiago, Chile; ^2^Faculty of Physical Culture Sciences, Universidad Autónoma de Chihuahua, Chihuahua, Mexico; ^3^Department of Psychology, Carleton University, Ottawa, ON, Canada; ^4^Department of Cognitive Science, Carleton University, Ottawa, ON, Canada

**Keywords:** early numeracy, home learning, home numeracy, children, numeracy activities, literacy activities, Mexico, socioeconomic status

## Abstract

We explored the home learning environments of 173 Mexican preschool children (aged 3–6 years) in relation to their numeracy performance. Parents indicated the frequency of their formal home numeracy and literacy activities, and their academic expectations for children’s numeracy and literacy performance. Children completed measures of early numeracy skills. Mexican parent–child dyads from families with either high- or low-socioeconomic status (SES) participated. Low-SES parents (*n* = 99) reported higher numeracy expectations than high-SES parents (*n* = 74), but similar frequency of home numeracy activities. In contrast, high-SES parents reported higher frequency of literacy activities. Path analyses showed that operational (i.e., advanced) numeracy activities were positively related to children’s numeracy skills in the high- but not in the low-SES group. These findings improve the understanding of the role of the home environment in different contexts and provide some insights into the sources of the variable patterns of relations between home learning activities and children’s numeracy outcomes. They also suggest that SES is a critical factor to consider in research on children’s home numeracy experiences.

## Introduction

Children’s early numeracy skills are strongly related to the development of their mathematical knowledge in the first few years of schooling (e.g., Jordan et al., 2009; Aunio and Niemivirta, 2010; LeFevre et al., 2010a; Martin et al., 2014). Because these individual differences in early numeracy knowledge precede children’s school entry (Duncan et al., 2007), researchers have identified the home learning environment as a potential source of some of this variability (Blevins-Knabe and Musun-Miller, 1996; LeFevre et al., 2009; Skwarchuk et al., 2014; Soto-Calvo et al., 2020a,b). Consistent with the view that home numeracy experiences are related to children’s numeracy preparation, parents’ reports of home numeracy activities are correlated with children’s early numeracy performance in many countries, including Canada (LeFevre et al., 2009; Skwarchuk et al., 2014), the United States (Blevins-Knabe and Musun-Miller, 1996; Huntsinger et al., 2016), Netherlands (Kleemans et al., 2012, 2013; Segers et al., 2015), Germany (Anders et al., 2012; Niklas and Schneider, 2014), Greece (LeFevre et al., 2010b; Manolitsis et al., 2013), and China (Pan et al., 2006; Huang et al., 2017). Early numeracy skills have also been found to be related to parental reports of literacy activities in the United Kingdom (Soto-Calvo et al., 2020a,b), the United States (Napoli and Purpura, 2018), and Germany (Anders et al., 2012). Thus, research suggests that the home numeracy and literacy experiences of children in North American, European, and Asian nations are related to the development of their early numeracy skills.

In Latin America, researchers have also identified the home numeracy environment as a correlate of children’s early numeracy skills (Becerra Orellana, 2016; del Río et al., 2017; de León et al., 2020; Susperreguy et al., 2020a,b). However, although they show that the parental reports of home numeracy activities predict children’s numeracy outcomes, the connections are weaker (Susperreguy et al., 2020b) than those reported in prior research (Skwarchuk et al., 2014). A model of home learning environment simultaneously including both literacy and numeracy to predict children’s numeracy outcomes has not been tested in Latin American countries.

The present research examines these relations in Mexico. The Mexican economy is one of the most inequitable among Latin American countries (Reyes et al., 2017), with 44% of the population living in poverty (Varela Llamas and Ocegueda Hernández, 2020). Mexico is ranked as number 74 in the Human Development Index (HDI) ranking, which is a measure of the development of a country that considers three dimensions – life expectancy, education (i.e., expected years of schooling and mean years of schooling), and gross national income per capita (United Nations Development Programme, 2020). With regards to education, Mexicans attain a mean of 8.8 years of schooling, which corresponds to some secondary education. These socioeconomic status (SES) inequalities may be linked to differences in children’s home learning environments, for example, parental educational backgrounds and access to educational resources, which might affect parental academic expectations and home activities (Davis-Kean et al., 2020). Thus, SES may be an important factor in the home learning experiences of Mexican children.

The goals of the present research are (a) to examine the home learning environment of children in Mexico, a Latin American country that has received little attention in this literature, and (b) to explore whether there are differences between low- and high-SES Mexican samples in the relations between the home learning environments and children’s numeracy outcomes.

### Contextual Factors in Early Numeracy Skills

According to the socio-cultural theory, children develop skills from their social and cultural contexts (Vygotsky, 1978; Rogoff, 2003). These contexts involve not only broader social institutions, such as schools, but also the formal and informal practices carried out by the primary caregivers in their day-to-day interactions with their children (Rogoff, 1990). For example, with respect to children’s learning, some research indicates that Latino parents engage in relatively few academic activities with their children given that they tend to rely on the school system to teach their preschool children the relevant academic concepts and skills (Goldenberg, 2001). Furthermore, when Latino parents do engage their children in academic activities, these are consistent or complementary with the teacher’s recommendations and assigned homework (Gonzalez et al., 2019; Sonnenschein and Dowling, 2019). In addition, Latin American families show some differences regarding access to resources, home learning experiences, and academic expectations, compared to parents from other countries (Strasser et al., 2016). Thus, early numeracy skills develop from an early age within the home learning environment, where parents provide a wide range of opportunities depending on their cultural context.

### The Home Numeracy Model

The influence of the learning environment on early numeracy skills has been explained by the Home Numeracy Model proposed by Skwarchuk et al. (2014). This model shows that parental academic expectations are linked to the frequency of engaging in home learning activities, which in turn predict children’s numeracy skills. Thus, parents who report higher expectations about their children’s achievement of academic benchmarks also engage in home learning activities with their children more frequently (Kleemans et al., 2012; Segers et al., 2015).

In addition to parental academic expectations, the home learning environment includes literacy and numeracy activities (i.e., home learning activities). These typically have been assessed with parental reports of how frequently they engage with their children in distinct types of activities that promote learning (e.g., Skwarchuk et al., 2014; Mutaf-Yıldız et al., 2018; Susperreguy et al., 2020b). These activities have been classified according to the nature and focus of the interactions. For literacy, activities that involve attention to print are referred to as code-based activities, whereas those in which the focus is on the meaning of the print are referred to as meaning-related activities (Sénéchal et al., 2017). For numeracy, activities have been classified as either mapping activities (i.e., basic numerical activities that link different number representations, such as naming or writing numbers), or operational activities (i.e., more complex number activities that involve manipulation of symbolic quantities; Susperreguy et al., 2020b). Of these learning activities, operational numeracy activities are related to children’s mathematics performance, as found by several studies (Mutaf-Yıldız et al., 2020).

Home numeracy and literacy activities are differentially related to children’s mathematics performance (Skwarchuk et al., 2014; Soto-Calvo et al., 2020a,b). For example, in a sample of families from the United Kingdom, Soto-Calvo et al. (2020a) found that parents’ reports of the frequency of mapping numeracy activities and code-based literacy activities were correlated with preschool children’s symbolic numeracy skills (i.e., counting, number transcoding, and calculation). However, only code-based literacy activities (not meaning-related activities) predicted unique variance in children’s symbolic numeracy skills. Soto-Calvo et al. (2020a) did not ask parents about more advanced numeracy activities (i.e., operational activities such as practicing calculations). In most other research on home numeracy activities, only operational activities (i.e., more advanced), were related to numeracy performance (Skwarchuk et al., 2014; del Río et al., 2017; Susperreguy et al., 2020a). For Chilean children (Susperreguy et al., 2020a), only operational activities predicted mathematical performance. Code-based activities did not predict unique variance in children’s numeracy outcomes. Thus, although both numeracy and code-based literacy activities may be correlated with children’s mathematical skills, the pattern of unique variance may depend on whether parents’ reports are collected about advanced early numeracy activities.

The Home Numeracy Model has been tested with some Latin American parents. Researchers have found that this model extends to Chilean children (Susperreguy et al., 2020b). However, to our knowledge this model has not been tested in Mexico. In addition, although Susperreguy et al. (2020b) controlled for SES in their Chilean sample, they did not directly assess whether there were any differential relations between home learning environments and children’s numeracy skills by SES. It is important to examine these relations in different SES groups given the large social disparities that exist in Mexico. These disparities may translate into different access to resources, distinct degrees of cognitive stimulation in children’s learning environments, and unequal provision of opportunities for children’s numeracy learning (Davis-Kean et al., 2020). Consequently, academic expectations and home learning activities may vary by SES, and thus the relation of home learning activities to children’s numeracy outcomes may also differ.

### Home Learning Environments of Latino Families

#### Academic Expectations and Home Activities

Parental academic expectations have been studied with Mexican immigrant families to the United States. Some studies showed that parents whose children had not started formal schooling did not expect them to understand early literacy concepts, and thus home activities (e.g., teaching letter or words) were not considered useful (Reese and Gallimore, 2000). In more recent studies including Mexican immigrants, parents saw themselves as having an important role in their children’s math learning and reported that they engaged in home numeracy activities (Sonnenschein et al., 2016; Galindo et al., 2019). These findings are consistent with a study conducted by Gonzalez et al. (2019) with native Mexican parents. Gonzalez et al. (2019) found that parents viewed themselves as contributors to their children’s early literacy skills. Thus, the evidence regarding Latino parents’ views of the role of home versus school experiences is mixed (Goldenberg et al., 2001; Gonzalez et al., 2019). Adding to the differential findings, in these studies expectations have been assessed in terms of educational aspirations and other beliefs that parents have in terms of child development and home learning, and not about specific benchmarks to be achieved during elementary school (c.f., Susperreguy et al., 2020b). The Home Numeracy Model includes particular numeracy and literacy milestones as its measure of academic expectations. Thus, it is important to explore academic expectations in parents from Mexico to better understand those home learning environments.

As predicted by the Home Numeracy Model, research has found that parental academic expectations are related to home learning activities (Skwarchuk et al., 2014; Segers et al., 2015; Kleemans et al., 2018; Susperreguy et al., 2020b). In two Chilean studies in which parents reported on the importance of numeracy benchmarks and frequency of home numeracy activities (del Río et al., 2017; Susperreguy et al., 2020b), parents’ numeracy expectations were related to their reported home numeracy activities, consistent with prior research (Skwarchuk et al., 2014).

Research with Latino immigrant parents in North America (Jung et al., 2012) and Latino parents living in Latin America (Susperreguy et al., 2007; Strasser and Lissi, 2009) showed that these parents engaged in fewer literacy activities than non-Latino North American parents. For example, half of the parents of Chilean kindergarten children did not read children’s books to them (Susperreguy et al., 2007). Access to books, educational resources, and materials is limited because of the high cost of books and the lack of public libraries, factors that may influence home literacy experiences for Latin American children (Strasser et al., 2017). Additionally, although immigrant Latina mothers living in the United States stressed the importance of math, they did not have a systematic plan of action for promoting their children’s skills (i.e., did not provide relevant home experiences), compared to Chinese immigrant mothers (Sonnenschein et al., 2018). Similarly, immigrant mothers from Mexico predominantly taught math concepts at a basic level whereas Chinese mothers were more likely to teach these concepts at an advanced level (Tamis-LeMonda et al., 2013). In sum, the home environments provided by Latino parents may be different than those provided by other parents in North American contexts.

#### Home Learning Activities and Children’s Numeracy Outcomes in Latin America

Researchers studying children’s home experiences in Latin America have focused on the relations between numeracy outcomes and numeracy experiences rather than on the relations between numeracy outcomes and literacy activities, as in Soto-Calvo et al. (2020a). In terms of home numeracy environments, del Río et al. (2017) compared reports of home numeracy activities of fathers and mothers, and correlations of those reports with the problem-solving skills of 180 Chilean kindergarteners. They found that the frequency of the numeracy activities reported by mothers was related to their children’s performance on a numeracy test. Similar links between numeracy activities and children’s numeracy outcomes were reported by Becerra Orellana (2016) for Ecuadorian children, by de León et al. (2020) for Uruguayan children, and by Susperreguy et al. (2020b) for a different sample of Chilean children. Moreover, Leyva (2019) found that Chilean parents provided moderate levels of math support to their Chilean children in a grocery game and that the degree of support predicted children’s gains in a problem-solving task. These studies suggest that similar relations exist between parents’ reports of home numeracy activities and their children’s performance as in North American and European studies.

Although most of the studies in Latin America have focused on the relations, within a single domain (i.e., literacy or numeracy), between home activities and children’s outcomes, few studies have assessed cross-domain relations between numeracy and literacy activities and children’s numeracy outcomes (e.g., Susperreguy et al., 2020b). Contrary to the results for children in the United Kingdom reported by Soto-Calvo et al. (2020a, b), Susperreguy et al. (2020b) found that only numeracy, not literacy, activities predicted Chilean children’s numeracy outcomes. Notably, Susperreguy et al. (2020b) assessed complex home numeracy activities (i.e., operational activities), whereas Soto-Calvo et al. (2020a, b) only assessed basic numeracy activities. In Susperreguy et al. (2020b), only operational activities predicted children’s skills (see also Skwarchuk et al., 2014). Thus, we did not expect to find cross-domain relations between literacy activities and children’s numeracy in Latin American contexts.

#### Home Learning Activities and Numeracy Outcomes of Latino Children in the United States

Research conducted with Latino families living abroad (mainly in the United States) suggests more variable relations between parents’ reports of home activities and children’s early skills for Latino families. Although Sonnenschein et al. (2016) found a significant relation between math activities and performance in a sample comprising low-income Latino-American immigrant and African-American families, in other studies that included Hispanic families, parents’ reports of math activities were *not* related to their children’s early numeracy skills (DeFlorio and Beliakoff, 2015; Missall et al., 2015; Leyva et al., 2017). Notably, those three studies assessed home numeracy experiences using different measures than the studies that have found positive links. Thus, the differential findings may not be related to the inclusion of Hispanic families in these studies but to the ways in which home numeracy experiences were assessed. In summary, there is a need for better understanding of the links between children’s home experiences and their early numeracy performance in families with roots in Latin American countries.

### Socioeconomic Status and Home Learning Environment

Socioeconomic status is related to many aspects of children’s academic and social development (Elliott and Bachman, 2018; Davis-Kean et al., 2020). SES is usually operationalized as parents’ educational attainment or as family income (Davis-Kean et al., 2020). Researchers interested in the home math environment have most often controlled statistically for SES (Hart et al., 2016; Mutaf-Yıldız et al., 2020). Other researchers have explored the home mathematics environment for low-SES families more directly (Jordan and Levine, 2009; Ramani et al., 2015). Results of these studies suggested that low-SES parents in the United States provided fewer opportunities for children to engage in complex math-related activities than high-SES parents, had fewer resources such as books or games (Starkey and Klein, 2008), and engaged in less math-related talk (Ramani et al., 2015). These studies support the view that SES is an important variable to consider in research on the home mathematics environment.

In Latin American countries, access to educational resources is unequal and parents are less likely to engage in activities that are common in other countries with access to more resources, such as shared book reading (see Strasser et al., 2016, for a review). Such factors may contribute to different patterns of parental activities in Latin American countries. In Mexico, given the vast inequities in SES, it is important to examine whether the patterns of relations between the home learning activities and children’s numeracy outcomes vary by SES. Thus, simply controlling for SES may not be sufficient for understanding the variability of home environments in Mexican families.

Findings with respect to parental academic expectations and SES are mixed. For example, DeFlorio and Beliakoff (2015) concluded that low-SES American parents have a less accurate understanding of the development of early academic capabilities in relation to their 3- and 4-year-old children’s skills than do parents from middle-SES backgrounds. In particular, low-SES parents underestimated the importance of mathematical benchmarks that were within children’s expected abilities according to their developmental stage and overestimated the importance of solving arithmetic problems which were beyond what would be expected. However, low-SES parents of 5- and 6-year-old children in Belgium reported significantly higher academic expectations than did high-SES parents (De Keyser et al., 2020). Similarly, for Chilean 4-year-old children, less-educated parents had higher academic expectations for their children than more-educated parents (Susperreguy et al., 2020b). However, Susperreguy et al. (2020b) did not compare parental academic expectations in different SES groups. The inconclusive findings on the relation between parental academic expectations and SES highlight the need to further explore academic expectations in other contexts with large SES disparities and compare the home learning environments in different SES groups.

### Current Study

Accordingly, the current study extends research on home learning environments and numeracy outcomes to Mexican families. We used a Home Learning Environment survey tested on Chilean families (Susperreguy et al., 2020b) to explore the home environments of children from low- versus high-SES communities in Mexico. We then assessed whether the patterns of relations between parents’ reports of learning activities at home and children’s numeracy performance varied by SES.

For our first hypothesis, we expected that low-SES parents would report higher academic expectations than high-SES parents (Hypothesis 1). This was based on the findings that low-SES parents have less accurate expectations for their children (DeFlorio and Beliakoff, 2015), and the negative association between SES and expectations in a Latin American study (Susperreguy et al., 2020b) using the same questionnaire as in the present study. Second, we expected that low-SES parents would report fewer activities (Starkey and Klein, 2008) than high-SES parents (Hypothesis 2). Third, we hypothesized that parents’ academic expectations would be associated with the frequency of engaging in home learning activities (Skwarchuk et al., 2014) in both SES groups (Hypothesis 3). Fourth, based on prior research (Skwarchuk et al., 2014; Susperreguy et al., 2020b) we hypothesized that parent’s reports of operational, but not mapping, home numeracy activities would be linked to children’s numeracy performance in both SES groups (Hypothesis 4). Fifth, we hypothesized that when numeracy activities were included in the model, neither code-based nor meaning-related (Susperreguy et al., 2020b) literacy activities would predict children’s numeracy outcomes in either SES groups (Hypothesis 5). Finally, given the expected SES differences in parental reports of home activities and academic expectations, we hypothesized that the strength of the associations between home learning activities and children’s numeracy outcomes would be stronger for the high-SES group than for the low-SES group (Hypothesis 6).

## Materials and Methods

### Participants

One hundred and seventy-three children and one of their parents participated in the study. Ninety-nine children (43 girls) were recruited from two public schools in very low-SES neighborhoods in a southern community of the State of Chihuahua (see Table 1 for descriptive information), whereas 74 children (34 girls) were recruited from four private schools in the city of Chihuahua. Mean age for high-SES children was 4 years and 8 months (*SD* = 11.06 months; range = 35 - 76 months), and mean age for low-SES children was 4 years and 9 months (*SD* = 9.52 months; range = 36–72 months). All children were monolingual Spanish speakers. Mothers’ education differed by SES. Eighteen mothers in the high-SES group, and four in the low-SES group did not respond to this question; however, the other 56 high-SES mothers were significantly more highly educated than their low-SES counterparts. The educational attainment of mothers from high-SES backgrounds ranged from “graduated from technical/applied college” to “have a postgraduate degree,” with a median of “graduated from university.” Low-SES mothers’ educational levels ranged from “less than high school” to “graduation from university,” with a median of “less than high school.” Mother’s education was higher for the high-SES group than for the low-SES group, *χ*^2^ (4, *N* = 151) = 110.06, *p* < 0.001.

**TABLE 1:** Descriptive statistics for low-SES and high-SES groups.

	Low-SES			High-SES			Independent *t*-test			
	*M*	*SD*	*N*	*M* (*SD*)	*SD*	*N*	*t*	*df*	*CI*s	Cohen’s *d*
Age (in months)	56.75	9.52	99	55.56	11.06	73	0.75	170	[−1.92, 4.29]	0.12
Numeracy expectations	0.27	0.91	87	–0.33	0.92	70	4.11***	155	[0.31, 0.89]	0.66
Literacy expectations	–0.03	0.88	87	0.03	1.08	70	–0.37	155	[−0.37, 0.25]	0.07
Mapping activities^a^	–0.07	1.00	92	0.08	0.84	71	–1.02	159.83	[−0.44, 0.14]	0.16
Operational activities	0.00	0.95	92	0.00	0.90	71	–0.06	161	[−0.30, 0.28]	0.00
Code-based activities	–0.15	0.98	91	0.19	0.90	71	−2.31*	160	[−0.64, −0.05]	0.36
Meaning-related activities	–0.22	0.95	91	0.29	0.82	71	−3.61***	160	[−0.79, −0.23]	0.57
Number comparison	13.69	5.08	87	14.41	4.92	70	–0.90	155	[−2.31, 0.86]	0.14
Cardinality	3.99	2.23	96	4.65	2.10	72	–1.96	166	[−1.33, 0.01]	0.30
Verbal counting^a^	15.84	11.66	96	20.76	18.21	72	−2.01*	113.36	[−9.78, −0.06]	0.32

^*a*^Adjusted *df* was used to correct for unequal variance. Descriptive statistics for academic expectations and home learning activities are factor scores. *p < 0.05 and ***p < 0.001.

### Materials

#### Parent Questionnaire

We used the same items as Skwarchuk et al. (2014) to assess parents’ academic expectations for children entering Grade 1 (see Supplementary Table A1 in Appendix A) and the frequency with which they engaged their children in home numeracy and literacy activities (see Supplementary Table A2 in Appendix A). The Spanish version of this questionnaire has been used and tested in other Latin American samples (see Jiménez Lira, 2016; Susperreguy et al., 2020b). Similar questions have been employed with parents in a range of countries, including the United States (Zippert and Ramani, 2017), Canada (LeFevre et al., 2009), Greece (LeFevre et al., 2010b), Germany (Anders et al., 2012), and China (Huang et al., 2017), supporting the use of these questions in a range of countries.

For numeracy, the questionnaire asked about mapping activities and operational activities. *Mapping activities* included five items describing activities in which children were encouraged to learn or practice number symbols (verbal or visual; Susperreguy et al., 2020b): singing number songs, recognizing digits, asking about quantities, reciting numbers, and indicating quantities with fingers. *Operational activities* included five items describing more complex tasks that involve manipulating numbers and/or quantities (Susperreguy et al., 2020b): doing mental math; weighing, measuring, or comparing quantities; learning simple sums; playing games involving counting, adding, or subtracting; and talking about time with clocks and calendars. For literacy, six items were code-based activities (i.e., activities that involve attention to print) and five were meaning-related items (i.e., the meaning of the print is the focus of the interaction) (Sénéchal et al., 2017). However, one of the meaning-related items (i.e., visiting the library for children’s books) showed a very low frequency (see Supplementary Table A2 in Appendix A) and thus only the other four items were considered in further analyses.

#### Numeracy Measures

The data for this study are part of a larger ongoing research project in which children complete motor skill assessments, literacy, and numeracy measures. We assessed numeracy skills by using three tasks that capture different aspects of early number knowledge: verbal counting, cardinality, and verbal number comparison.

##### Verbal counting

Children were asked to count as high as possible. Their highest verbal count without any errors was used as the measure of performance. The reported test-retest reliability for this measure with 3- and 4-year-old Canadian children in prior research was *r* = 0.580 (Dunbar et al., 2017).

##### Cardinality

Children completed a Give-N task, where they were asked to give a puppet a set of 1 to 6 foam cubes. First, the child was asked to provide 1 cube. If the child succeeded, he or she was asked for 2; if the child failed to correctly provide 2 cubes, the experimenter again asked for 1; if the child was successful, the experimenter asked for 2 cubes again; and if the child succeeded, the experimenter would ask for the next number (i.e., 3). The task continued until the child had at least two successes at a given number or the child reached the highest number assessed (i.e., 6) (Sarnecka and Carey, 2008). The score was the highest set size with two correct trials. Cronbach’s α for the pass/fail score of each number 1–6 for low- and high-SES were 0.911 and 0.932, respectively.

##### Verbal number comparison

The purpose of this task was to assess children’s ability to mentally compare two number words and determine which represented the greater quantity. Children completed a total of 20 trials in which they were told that the puppets Dolly the sheep and Belle the cow had gone shopping for fruits and vegetables. Children were told the amount of food each one had bought (e.g., “Dolly bought five apples and Belle bought eight apples”), without showing any visual stimuli, and the experimenter would then ask the child to point to the puppet that had bought more fruit (e.g., “Who bought more apples?”). All the numbers from 1 to 9 were assessed. No feedback was provided as to the correctness of the answer, but children were verbally encouraged at all times. The score on this task was the total number of correct responses. Cronbach’s α for the twenty items for the low- and high-SES were 0.925 and 0.890, respectively.

### Procedure

Parents were recruited from schools, serving either low-SES (public) or high-SES (private) communities. The public schools were classified as low-SES according to the information obtained from the Educational Services of the State of Chihuahua, who provided a list of schools from which participants were selected. To obtain permission to test children in the public schools, letters were sent to the director of the Chihuahua Board of Preschool Education, to the supervisor of the school district, and to the school principal explaining the nature of the study. For the private schools, a letter explaining the nature of the study was provided to the school principal of several institutions (by convenience). Once authorization was granted, we sent the informed consent form and the parent questionnaires home to the parents by asking each of the teachers to hand them out. Parents were asked to return the questionnaires in a sealed envelope (which was included) in order to ensure confidentiality. Children whose parents provided informed consent were asked whether they wished to participate. Prior to testing, children gave verbal assent. After completing the tasks children were thanked for their participation and awarded a sticker.

Testing took place in a quiet area of the school. A trained experimenter visited the children twice within one week to complete the measures. The tasks were presented to the children in one of five fixed orders, which was done to avoid task order effects. Testing of all children took four months.

### Analytical Strategy

First, we used Principal Axis Factoring analyses (PAFs) to explore the structure of the components of the questionnaire (i.e., expectations, numeracy activities, and literacy activities). Second, we compared the low- and high-SES groups on parental expectations (Hypothesis 1) and reported home activities (Hypothesis 2), using independent *t*-tests. For these analyses, we used SPSS (version 25). Third, we used multi-group path analyses to assess the associations among parental expectations and home learning activities by SES (Hypothesis 3), and to test the relations among children’s numeracy performance and parents’ reports of their home activities in the two SES groups. Thus, we tested whether home learning activities predicted numeracy performance in both SES groups (Hypotheses 4 and 5); and we examined whether the strength of the associations differed by the SES of the families (Hypothesis 6). For the multi-group analyses, M*plus* 8 (version 1.6) was used (Muthén and Muthén, 1998-2012). Data were missing for some children on the six home numeracy factors (ranging from 5.8 to 9.2% across children) and on the numeracy outcome (9.2%). Little’s test for missingness showed that data were missing completely at random, *χ*^2^(25) = 35.23, *p* = 0.084. Thus, the models were estimated by a full information maximum likelihood method, which uses all available information to estimate the model. Furthermore, children’s age was included as a control variable; however, no differences by gender were found for parental numeracy and literacy expectations, or for home learning activities (i.e., operational, mapping, code-based and meaning-related activities) nor for the numeracy outcome, all *p*s > 0.05, thus, gender was not included in further analyses.

## Results

### Factor Analyses of the Parent Questionnaire

Given that the low- and high-SES groups differ substantially on most of the item scores (see Supplementary Tables A1, A2 in Appendix A), we standardized the item scores within each group to reduce the possibility that correlations would be inflated due to scaling differences. Principal Axis Factoring analyses with oblique rotation using the Promax procedure with Kaizer Normalization were then conducted for each of the three scales of the home questionnaire (i.e., parental academic expectations, numeracy activities, and literacy activities). Note that in many previous studies, orthogonal rotation was chosen to create uncorrelated factors. In contrast, oblique rotation was used in the current study because preliminary analyses indicated that factors were correlated above 0.30 (Field, 2013). The general patterns of correlations across items were similar between the low- and high-SES parents, thus we proceeded with a single factor analysis based on the data for all participants for each of the questionnaire scales. For parental expectations, the factor analyses resulted in two components (numeracy and literacy expectations) that accounted for 57.8% of the variance (see Supplementary Table B1 in Appendix B). The factor analyses for the numeracy activities resulted in two components (mapping and operational activities) that accounted for 44.0% of the variance (see Supplementary Table B2 in Appendix B). For literacy activities, as shown in Supplementary Table B3 (Appendix B), factor analyses resulted in two components (code-based and meaning-related activities) that accounted for 52.7% of the variance. The factor scores from each analysis were saved and used in the subsequent analyses. The reliability for the numeracy and literacy expectations were Cronbach’s α = 0.886 (0.879), 0.908 (0.943), respectively for the low-SES (high-SES) group. For the home learning activities, Cronbach’s α for mapping activities, operational activities, code-based activities, and meaning-related activities were 0.830 (0.859), 0.814 (0.821), 0.908 (0.885), and 0.775 (0.834), respectively for the low-SES (high-SES) group.

### Similarities and Differences of Parental Expectations and Activities by SES

Descriptive statistics and group comparisons (high- vs low-SES) for the variables can be found in Table 1. For parental academic expectations and home learning activities, factor scores are shown. Detailed item-level information about parent’s reports of academic expectations and home learning activities by SES is available in Supplementary Tables A1 and A2 in Appendix A. Correlations among the variables are found in Table 2.

**TABLE 2:** Correlations of factor scores for expectations and home numeracy and literacy activities and child outcomes.

	1	2	3	4	5	6	7	8
1. Age	–	−0.152	−0.058	−0.090	0.066	0.424***	0.241	0.769***
2. Numeracy exp.	−0.063	–	0.693***	0.288*	0.323**	0.325**	0.264*	0.047
3. Literacy exp.	−0.082	0.746***	–	0.329**	0.298*	0.412***	0.239*	0.151
4. Mapping	0.167	0.039	0.292**	–	0.748***	0.574***	0.666***	0.010
5. Operational	0.330**	0.139	0.309**	0.757***	–	0.575***	0.605***	0.294*
6. Code	0.292**	0.068	0.248*	0.754***	0.718***	–	0.757***	0.486***
7. Meaning	0.200	0.096	0.261*	0.722***	0.803***	0.843***	–	0.292*
8. Numeracy factor	0.637***	0.019	0.034	0.114	0.173	0.192	0.114	–

Low-SES group below the diagonal, high-SES group above the diagonal. *p < 0.05, **p < 0.01, and ***p < 0.001.

#### Parents’ Numeracy and Literacy Expectations

We hypothesized that low-SES parents would report higher academic expectations than high-SES parents (Hypothesis 1). As shown in Table 1, low-SES parents reported higher numeracy expectations than high-SES parents, *t*(155) = 4.11, *p* < 0.001, *d* = 0.66. Specifically, the low-SES parents rated all but the know simple sums item as more important than did the high-SES parents (see Supplementary Table A1 in Appendix A), even though several of these are unrealistic benchmarks for most children to achieve before Grade 1. However, there were no differences in their literacy expectations factor, *t*(155) = −0.37, *p* > 0.05, *d* = 0.07. These results provide partial evidence in favor of our hypothesis: Low-SES parents reported higher numeracy, but not literacy, expectations than high-SES parents.

#### Parents’ Reports of Numeracy and Literacy Activities

We expected that low-SES parents would report engaging in activities less frequently than high-SES parents (Hypothesis 2). Our findings support the hypothesis for the literacy activities, but not for numeracy (see Table 1 and Supplementary Table A2 in the Appendix A). High-SES parents reported higher frequency of both code-based, *t*(160) = −2.31, *p* < 0.05, *d* = 0.36, and meaning-related activities, *t*(160) = −3.61, *p* < 0.001, *d* = 0.57, compared to low-SES parents. In contrast, no differences between high- and low-SES parents were found in reported frequencies of mapping and operational activities.

### Multi-Group Path Analyses

Multi-group path analyses were conducted to assess the links between parental academic expectations and home learning activities (Hypothesis 3), and between parental reports of numeracy (Hypothesis 4) and literacy (Hypothesis 5) activities and children’s numeracy performance, and whether the strength of these relations would vary by SES (Hypothesis 6).

For data reduction, a principal component analysis (PCA) was used to create a numeracy factor using the three measures: number comparison, cardinality, and counting (factor loadings of 0.89, 0.84, and 0.83, respectively). The factor accounted for 72.9% of the variance in these measures. This factor score was saved and used in the subsequent analyses.

Prior to the modeling, given the high correlations between the numeracy and literacy expectations, and among the four types of home activities (i.e., mapping, operational, coded-based, and meaning-related, see Table 2), we examined multicollinearity among these variables. A variance inflation factor (VIF) of 5 or more, and/or a tolerance of 0.2 or less, indicates that there is multicollinearity among the variables (Belsley, 1991; Field, 2013). In the present study, in initial analyses, multicollinearity was detected for the meaning-related activities for the low-SES group (VIF = 6.67; tolerance = 0.150), suggesting that meaning-related activities were not distinguishable from the other home learning activities for this group of parents. Moreover, there were no significant correlations between meaning-related activities and numeracy skills for either low- or high-SES groups (see Table 2). Because relations between meaning-related activities and children’s numeracy have not been reported in previous research, we did not hypothesize that meaning-related activities would be linked to children’s numeracy performance. Thus, we removed meaning-related activities from the model.

Although multicollinearity was not detected for mapping activities, the bivariate correlation between mapping and operational activities was over 0.70 for both SES groups, which might affect the stability of the findings. Because mapping activities was also uncorrelated with the numeracy factor, and we did not hypothesize a link, we also excluded mapping activities from the model.

The initial analyses in which both numeracy and literacy expectations were included in the same model showed that there was a strong suppressor relation among expectations and activities, which distorted the interpretation of the pattern of results (Ludlow and Klein, 2014). These patterns were probably a function of the high correlations among these variables, especially for the expectations. Thus, in the following analyses, we tested two separate models: one involving numeracy expectations and one involving literacy expectations.

The multi-group path analysis involves testing the cross-group invariance, that is, the assumption that the structural parameters are statistically different between the two groups. In particular, we compared nested models: 1) an unconstrained model in which the coefficients for each of the paths were estimated freely for each group, and 2) a constrained model in which the coefficients were set to be equal across groups. Note that the models for the full sample without the grouping variable are shown in Appendix C (Supplementary Figure C1).

**FIGURE 1:**
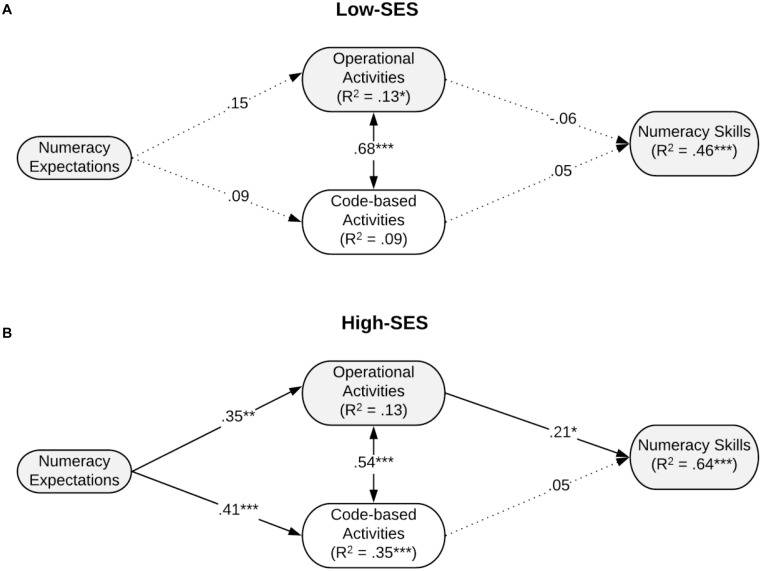
Final path model shows relations among numeracy expectations, home learning activities, and children’s outcome for low SES group (**A**: *n* = 99) and high SES group (**B**: *n* = 74) controlling for child’s age. The *R*^2^ values shown include variance predicted by the control measure. The numbers on the arrows are the standardized coefficients. Dashed lines present no significant paths. *p < 0.05, **p < 0.01, and ***p < 0.001.

For the model that included numeracy expectations, the unconstrained model had an excellent fit to the data, *χ*^2^(2) = 1.15, *p* = 0.564, SRMR = 0.01, CFI = 1, RMSEA = 0 (90% CI = [0, 0.18]) whereas the fully constrained model had a poor fit to the data, *χ*^2^(10) = 22.36, *p* = 0.013, SRMR = 0.07, CFI = 0.95, RMSEA = 0.12 (90% CI = [0.05, 0.19]). Accordingly, the constrained model had a statistically poorer fit to the data than the unconstrained model, Δ*χ*^2^(8) = 21.21, *p* = 0.007, suggesting that the path coefficients were different for the low-versus high-SES children. These models are shown in Figure 1.

**FIGURE 2:**
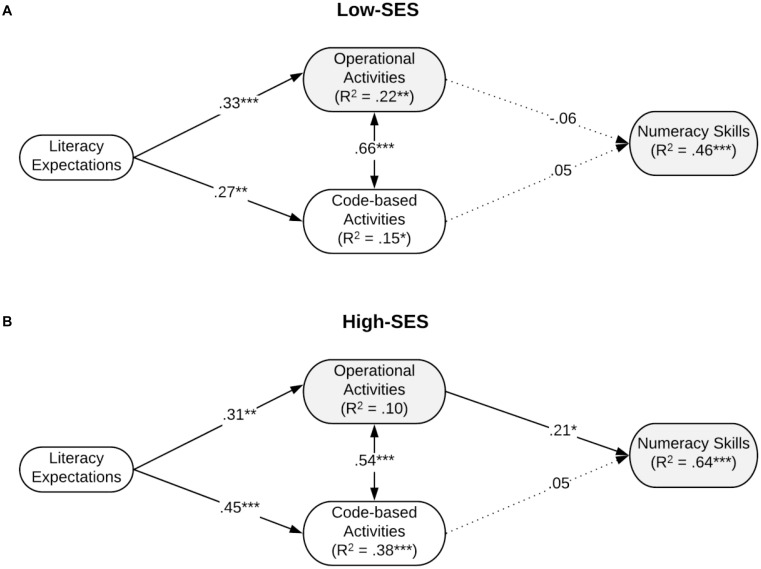
Final path model shows relations among literacy expectations, home learning activities, and children’s outcome for low SES group (**A**: *n* = 99) and high SES group (**B**: *n* = 74) controlling for child’s age. The *R*^2^ values shown include variance predicted by the control measure. The numbers on the arrows are the standardized coefficients. Dashed lines present no significant paths. *p < 0.05, **p < 0.01, and ***p < 0.001.

Similarly, for the model that included literacy expectations, the unconstrained model had an excellent fit to the data, *χ*^2^(2) = 2.94, *p* = 0.230, SRMR = 0.02, CFI = 1, RMSEA = 0.07 (90% CI = [0, 0.24]) whereas the fully constrained model had a poor fit to the data, *χ*^2^(10) = 21.30, *p* = 0.019, SRMR = 0.06, CFI = 0.96, RMSEA = 0.11 (90% CI = [0.04, 0.18]). Accordingly, the constrained model had a statistically poorer fit to the data than the unconstrained model, Δ*χ*^2^(8) = 18.36, *p* = 0.019, suggesting that the path coefficients were different for the low-versus high-SES children. These models are shown in Figure 2.

In all of the models, we controlled for child’s age (not shown in figures for readability). In terms of the associations of the control variable with the home learning activities, for the low-SES group, age was a significant predictor of operational activities (*β* = 0.34, *p* < 0.001; *β* = 0.36, *p* < 0.001, for the numeracy and literacy expectation models respectively) and code-based activities (*β* = 0.29, *p* < 0.01; *β* = 0.30, *p* < 0.01, for the numeracy and literacy expectation models, respectively). For the high-SES group, age was only related to code-based activities (*β* = 0.49, *p* < 0.001; *β* = 0.46, *p* < 0.001, for the numeracy and literacy expectation models respectively), but not to operational activities (*β* = 0.12, *p* > 0.05; *β* = 0.09, *p* > 0.05, for the numeracy and literacy expectation models, respectively). Age was a significant predictor of numeracy skills for both low- (*β* = 0.68, *p* < 0.001) and high- (*β* = 0.73, *p* < 0.001) SES groups in both models.

In summary, low-SES parents reported more home numeracy and code-based home literacy activities for older than for younger children. High-SES parents reported more code-based activities for older than for younger children.

#### Relations Between Academic Expectations and Home Learning Activities

The paths from parental academic expectations to home activities differed across groups for the model that included numeracy expectations (see Figure 1). We hypothesized that parents’ academic expectations for children’s performance would be related to their reports of home learning activities in both SES groups (Hypothesis 3). However, for the low-SES group, we found that numeracy expectations were unrelated to either operational or code-based activities whereas literacy expectations were positively related to both operational and code-based activities (see Figure 2). For the high-SES group, both numeracy and literacy expectations were significantly related to both operational and code-based activities. In summary, although there were some differences in the patterns of relations by SES, Hypothesis 3 was partially supported in the low-SES group, whereas full support was provided for the high-SES group.

#### Relations Between Learning Activities and Children’s Numeracy Performance

We hypothesized that parents’ reports of operational numeracy (Hypothesis 4) but not code-based literacy or meaning-related literacy (Hypothesis 5) activities would be correlated with children’s numeracy performance in both SES groups. As shown in Figure 1 and consistent with our hypothesis, for the high-SES group, operational activities were positively related to numeracy skills (*β* = 0.21, *p* < 0.05). For the low-SES group, however, none of the home learning activities were related to numeracy skills. As expected, code-based activities were not related to numeracy skills in either group.

With regards to Hypothesis 6, the unconstrained models described above showed significantly better fit than the constrained models, indicating that the path coefficients differed between low- and high-SES groups.

In summary, these findings provide support for Hypothesis 4 (i.e., operational activities were positively related to children’s numeracy) and for Hypothesis 5 (i.e., code-based activities were not related to the outcome) for the high-SES group. For the low-SES group, there were no significant relations between parents’ reports of home activities and numeracy outcomes. Overall, in support of Hypothesis 6, the relations among the variables (expectations, activities, and the numeracy outcome) were different for the SES groups.

## Discussion

We explored parents’ academic expectations, parents’ reported home learning activities, and their links with early numeracy scores of low- and high-SES Mexican children. Our findings were generally consistent with prior research, showing different patterns of results for low- and high-SES parents in terms of their numeracy expectations (De Keyser et al., 2020) and literacy activities (Neumann, 2016; Ergül et al., 2017). Moreover, parents’ reports of the frequency of engaging in numeracy activities were related to children’s numeracy performance, but only in the high-SES group. These results add to the emerging literature on home learning environments in Latin America (Susperreguy et al., 2020a), and expand our understanding of the relations among SES, home numeracy activities, and children’s numeracy performance to Mexican families (Mutaf-Yıldız et al., 2020).

### Expectations for Numeracy and Literacy Achievement

Low-SES parents rated numeracy expectations as more important than did the high-SES parents. Overall, high-SES parents’ ratings of the importance of the numeracy benchmarks appear to be more closely calibrated to children’s skills (see Supplementary Table A1; DeFlorio and Beliakoff, 2015; Susperreguy et al., 2020b). For example, low-SES parents rated being able to “read printed numbers to 100,” “count to 1,000,” and “know multiplication” higher than high-SES parents. These skills are unlikely to be achieved by most children before starting Grade 1. The relatively high numeracy expectations of low-SES Mexican parents might indicate that their expectations are not informed by knowledge of children’s skills and their development (DeFlorio and Beliakoff, 2015) nor by their beliefs about the role of home experiences in children’s education (Reese et al., 1995; Goldenberg, 2001). Alternatively, the higher expectations of low-SES parents might reflect their high aspirations for their children. Research which explores the interpretations of these items by low-SES parents would help to illuminate these differences.

### Home Numeracy and Literacy Activities

We hypothesized that low-SES parents would report less frequent engagement in numeracy and literacy activities compared to high-SES parents. However, there were no SES differences in parents’ reported frequency of engaging in numeracy activities. This finding might indicate that both groups of parents are influenced by schooling and specifically, by the activities that teachers suggest or prescribe (Perry et al., 2008). Ongoing research including interviews with Mexican parents support this interpretation: the majority of the participating parents reported that they obtained most of the information on numeracy activities from their children’s teacher (Authors, manuscript in preparation), rather than from other sources.

In contrast, for literacy, high-SES parents reported a higher frequency of engaging in code-based and meaning-related activities than low-SES parents. These differences across SES groups in literacy activities might reflect different levels of knowledge and/or parents’ differential access to appropriate resources. High-SES families have more educational materials at home (Davis-Kean et al., 2020), a pattern that has also been reported in research with Latin American families (Strasser et al., 2017) and they may use these resources to facilitate home literacy activities. For example, high-SES parents may use children’s books to engage children, asking them to define words or point to letters or words as the parent reads. Low-SES parents, in contrast, may have only adult-level reading materials available, or possibly none other than what children need for school. Frequency of shared activities, on this view, may depend both on the resources that parents have available, as well as on their knowledge or beliefs about what types of activities are important for early learning.

### Academic Expectations and Home Learning Activities

Prior work shows that parents’ academic expectations are related to the reported frequency of engaging in home activities with their children (LeFevre et al., 2002; Martini and Sénéchal, 2012; Susperreguy et al., 2020b). In the present research, we found that for low-SES parents, literacy, but not numeracy, expectations were related to their reports of the frequency of home learning activities. In contrast, for high-SES parents both types of academic expectations were related to home learning activities.

An association between parents’ numeracy expectations and their numeracy activities has been found in previous work from various countries (LeFevre et al., 2002; Skwarchuk et al., 2014; del Río et al., 2017; Susperreguy et al., 2020b). In the present research, we found that parents’ numeracy expectations were unrelated to home learning activities for the low-SES group and that their expectations were generally higher than those of high-SES parents. However, these high expectations of the low-SES parents did not translate into a higher frequency of engaging in numeracy activities. This pattern suggests that, for the low-SES parents, reporting high expectations could reflect a social desirability bias. Alternatively, these parents may have a narrow understanding of early numeracy benchmarks needed to succeed in Grade 1. Galindo et al. (2019) found that Latina immigrant mothers to the United States, mostly from El Salvador, viewed mathematics as involving mainly arithmetic. They did not have a broad understanding of other aspects of mathematics that children learn in school. In the present study, the low-SES parents might not have an extensive knowledge of early numeracy and thus they rated all benchmarks as relatively important.

In contrast, for the high-SES group, numeracy and literacy expectations were positively related to both operational and code-based activities, as found in prior research (Skwarchuk et al., 2014). Thus, parents who had a more accurate understanding of the numeracy and literacy skills needed for school reported engaging in more frequent home activities that promote learning.

The differential findings between SES groups in the relations among numeracy and literacy expectations and home learning activities need to be further studied in Mexican and Latin American families in order to better understand the relations between SES and home learning experiences of children and their parents.

### Home Learning Activities and their Relation to Early Numeracy Skills

We hypothesized that parents’ reports of the frequency of home numeracy activities would be related to children’s numeracy performance in both SES groups, although we expected to find differences in the strength of the associations. This relation has been found in many, but not all, published studies (see Elliott and Bachman, 2017; Mutaf-Yıldız et al., 2020, for reviews). In the present study, there were no significant associations between the frequency of home numeracy activities and the numeracy performance of the low-SES children. In contrast, numeracy activities were related to the numeracy skills of high-SES children, as was found in prior research with Chilean children (del Río et al., 2017; Susperreguy et al., 2020b). del Río et al. (2017) included families from low- and high-SES backgrounds but they did not assess the differences in the relations between mother’s reported activities and children’s performance by SES. Moreover, most children in Susperreguy et al. (2020a) were from middle- to high-SES families. Thus, more systematic evaluation of the relations among children’s home numeracy environments, numeracy skills, and SES is clearly needed.

The lack of significant relations between the home activities and numeracy outcomes for the low-SES group in the current research suggests that the socioeconomic background of families is a key factor in understanding the links between home activities and children’s outcomes in Mexico. Studies with low-SES groups did not find correlations between parents’ reports of home numeracy activities and their children’s skills (e.g., DeFlorio and Beliakoff, 2015; Leyva et al., 2017). Various factors may lead to differential results for low-SES groups. First, differences in economic and educational conditions might influence the type of educational materials and learning opportunities provided by parents from different SES milieu (Starkey and Klein, 2008; Davis-Kean et al., 2020). These diverse conditions might translate to differential patterns of performance in low- and high-SES children. Second, the results could be related to parental knowledge of children’s skills and how to foster them (Zippert and Ramani, 2017). For example, low-SES parents may not have an understanding of children’s developmental stages (DeFlorio and Beliakoff, 2015), resulting in a lack of differentiation of home learning activities as a function of children’s age or accomplishments. Third, low-SES parents might engage in other activities not captured by the current assessment that was focused on formal activities. Low-SES parents could involve their children in activities linked to their everyday experiences, which they might not conceptualize as math (Civil et al., 2020). These activities may be more directly related to children’s numerical knowledge than the activities proposed in the questionnaire used in this study, which were all formal activities where parents had the intention to teach children about numbers.

In the present research we hypothesized that code-based activities would not be linked to children’s numeracy skills. In accord with this hypothesis but contrary to the findings by Soto-Calvo et al. (2020a, b), we did not find a link between code-based activities and children’s performance. Importantly, Soto-Calvo et al. (2020a, b) focused on basic numeracy activities and did not include operational activities in their model. Our results clearly show that only operational activities (i.e., relatively more complex) are related to children’s numeracy performance (see also Skwarchuk et al., 2014; Susperreguy et al., 2020a,b). Thus, when code-based literacy and operational numeracy activities are considered together, only operational activities were significantly related to children’s numeracy performance. We expect that code-based activities would be related to children’s early literacy performance (Sénéchal and LeFevre, 2014; Sénéchal et al., 2017). Thus, the present results are consistent with the view that the frequencies of home activities reported by parents are most closely linked to within-domain skills (Skwarchuk et al., 2014; Susperreguy et al., 2020b).

### Limitations and Future Directions

The current research has some limitations that should be considered in interpreting the findings. First, future studies should include alternative methods of data collection, such as interviews (Cahoon et al., 2017; Galindo et al., 2019), to understand differences in the experiences of children in low- versus high-SES families. These studies would help delineate the nature of parents’ academic expectations, identify variation in the quality of the activities, and therefore increase our understanding of the ways in which parents’ home activities are linked to children’s early mathematical skills in diverse SES groups (Ginsburg et al., 2012). Second, the study was not longitudinal and thus does not allow us to test the direction of the associations between home learning environments and children’s outcomes. Longitudinal studies that include assessments of the home learning environments and children’s numeracy performance at various time points are necessary to provide a clearer picture of the directionality of the links (Silinskas et al., 2020). Third, the current study used PCA and multi-group path analyses separately. Future studies with larger samples could test a multi-group SEM model, thereby testing the measurement invariance assumption of the latent home numeracy constructs between low- and high-SES groups. Fourth, we did not control for other cognitive skills that are relevant for children’s numeracy performance, such as executive functions (Blair and Razza, 2007) or children’s intelligence (Niklas and Schneider, 2014). Skwarchuk et al. (2014) controlled for spatial working memory in their work, whereas Kleemans et al. (2012) included a measure of working memory to account for child and family factors when predicting early numeracy skills. Beyond simply accounting for variability, home activities might also influence children’s cognitive skills and thus confound the role of domain-general and domain-specific influences on children’s academic skills.

Despite the limitations, our results are important for several reasons. This is the first study exploring the home learning environments and numeracy outcomes for Mexican children. We provide descriptive information on the similarities and differences in parents’ academic expectations and home activities in Mexican families by SES. Moreover, SES emerged as a critical moderating variable, suggesting that it should be closely examined in future work on the home numeracy environment.

## Conclusion

In conclusion, numeracy expectations were differentially associated with the frequency of home learning activities between the low- and high-SES groups. Significant associations were found between home numeracy activities and numeracy skills for Mexican children in high- but not low-SES families. Thus, high-SES families showed similar patterns of relations between parents’ reported numeracy activities and children’s numeracy performance as were found in other studies, whereas low-SES families did not. Most other research was done either with samples of predominately middle-class families (e.g., Skwarchuk et al., 2014; Susperreguy et al., 2020b) or with samples of low-SES families (Leyva, 2019). The present research highlights the need to consider the role of socioeconomic status as a moderator of the relations between home numeracy environment and children’s developing skills.

## Data Availability Statement

The raw data supporting the conclusions of this article will be made available by the authors, without undue reservation.

## Ethics Statement

The present study was reviewed and approved by Comité de Ética en Investigación de la Facultad de Medicina y Ciencias Biomédicas de la Universidad Autónoma de Chihuahua. Written informed consent to participate in this study was provided by the participants’ legal guardian/next of kin.

## Author Contributions

MS wrote the manuscript and developed the theoretical framework for the study. CJ designed and implementation of the study, supervised the data collection and analyses, interpretation of the results, and contributed to the manuscript writing. CX contributed to the data analyses, interpretation of the results, manuscript writing, and design of the figures. J-AL contributed to data analyses, interpretation of the results, manuscript writing, and to the theoretical framework for the study. HB contributed in design, implementation, and supervision of data collection, and contribution to the data analyses for the study. EB and MO contributed in design, implementation, and supervision of data collection for the study. All authors contributed to the article and approved the submitted version.

## Conflict of Interest

The authors declare that the research was conducted in the absence of any commercial or financial relationships that could be construed as a potential conflict of interest.
