# Supplementary material for: Home Learning Environments of Children in Mexico in Relation to Socioeconomic Status
Source: Front Psychol. 2021 Mar 19;12:626159. doi: 10.3389/fpsyg.2021.626159 (PMC8017273; doi:10.3389/fpsyg.2021.626159)
Supplement: Supplementary file 1 [file Table_1.docx]

Supplementary Material

# Appendix A. Detailed Information about Parents’ Reports of Academic Expectations and Home Activities by SES

**Table A.1**

*Academic Expectations by Socioeconomic Status (SES)*

|  | Low-SES | | High-SES | | *t* | *df* | *p* | *Cohen’s* *d* |
| --- | --- | --- | --- | --- | --- | --- | --- | --- |
|  | *M* | *SD* | *M* | *SD* |  |  |  |  |
| Numeracy |  |  |  |  |  |  |  |  |
| Count to 10^a^ | 3.20 | 0.86 | 3.46 | 0.83 | -2.00 | 160 | .048 | .31 |
| Count to 100 | 2.53 | 1.00 | 2.14 | 1.12 | 2.33 | 159 | .021 | .37 |
| Read printed numbers up to 100 | 2.42 | 1.05 | 1.85 | 1.16 | 3.20 | 160 | .002 | .52 |
| Know simple sums (e.g., 2+2) | 2.60 | 1.04 | 2.44 | 1.19 | 0.88 | 160 | .378 | .14 |
| Count to 1000 | 1.70 | 1.21 | 1.00 | 1.04 | 3.91 | 160 | <.001 | .62 |
| Know multiplying (e.g., 2x6) | 1.96 | 1.26 | 0.99 | 1.10 | 5.15 | 160 | <.001 | .82 |
| Literacy |  |  |  |  |  |  |  |  |
| Know some alphabet letters | 3.01 | 0.84 | 3.38 | 0.90 | -2.67 | 160 | .008 | .43 |
| Print his/her name | 3.34 | 0.79 | 3.37 | 1.02 | -0.16 | 162 | .875 | .03 |
| Know all letters of the alphabet | 2.77 | 1.13 | 2.83 | 1.15 | -0.33 | 161 | .742 | .05 |
| Print all letters of the alphabet | 2.64 | 1.14 | 2.61 | 1.21 | 0.19 | 161 | .848 | .03 |
| Read a few words | 2.65 | 0.94 | 2.89 | 1.17 | -1.45 | 160 | .150 | .23 |
| Read simple picture books | 2.47 | 1.05 | 2.21 | 1.34 | 1.37 | 161 | .174 | .22 |
| Read chapter books^a^ | 2.10 | 1.16 | 1.46 | 1.22 | 3.37 | 160 | .001 | .83 |

*Notes.* Parents responded to the question: *How important is it for your child to be able to achieve the following benchmarks before starting grade 1?* On a five point Likert scale: 0 - unimportant, 1 - neither important nor unimportant, 2 - important, 3 - very important, and 4 - extremely important. ^a^This question was not included in any analyses given its low variability/frequency in both groups. *N*s for low-SES = ranged from 90-93, high-SES = ranged from 70-71.

**Table A.2**

*Home Learning Activities by Socioeconomic Status (SES)*

|  | Low-SES | | High-SES | | *t* | *df* | *p* | *Cohen’s* *d* |
| --- | --- | --- | --- | --- | --- | --- | --- | --- |
|  | *M* | *SD* | *M* | *SD* |  |  |  |  |
| Numeracy Mapping Activities |  |  |  |  |  |  |  |  |
| I teach my child to recognize printed numbers | 2.62 | 1.15 | 2.86 | 1.02 | -1.41 | 163 | .161 | .22 |
| I ask about quantities (e.g., how many spoons) | 2.59 | 1.25 | 2.79 | 1.01 | -1.16 | 162.12 | .249 | .18 |
| I help my child to recite numbers in order | 2.83 | 1.18 | 3.07 | 0.93 | -1.42 | 163 | .158 | .23 |
| We sing counting songs | 2.24 | 1.43 | 2.32 | 1.26 | -0.37 | 163 | .712 | .06 |
| I encourage the use of fingers to indicate how many | 2.70 | 1.27 | 2.70 | 0.98 | -0.12 | 162.93 | .990 | .00 |
| Numeracy Operational Activities |  |  |  |  |  |  |  |  |
| I help my child learn simple sums | 1.50 | 1.31 | 1.37 | 1.34 | 0.64 | 163 | .521 | .10 |
| I encourage my child to do math in his or her head | 1.76 | 1.47 | 1.63 | 1.49 | 0.56 | 162 | .578 | .09 |
| We talk about time with clocks and calendars | 1.48 | 1.45 | 1.83 | 1.30 | -1.59 | 159.03 | .113 | .25 |
| I help my child weigh, measure, and compare quantities | 1.30 | 1.38 | 1.46 | 1.17 | -0.82 | 163 | .412 | .13 |
| We play games that involve counting, adding or subtracting | 2.10 | 1.38 | 1.89 | 1.30 | .0.98 | 162 | .330 | .16 |
| Literacy Code-based Activities |  |  |  |  |  |  |  |  |
| I help my child read words | 1.63 | 1.44 | 2.38 | 1.36 | -3.41 | 163 | .001 | .54 |
| I ask my child to point to letters/words when we read | 1.76 | 1.43 | 2.07 | 1.40 | -1.41 | 163 | .160 | .22 |
| I teach my child to recognize printed letters | 2.50 | 1.25 | 2.90 | 1.06 | -2.23 | 160.88 | .027 | .35 |
| I help my child print words | 2.23 | 1.36 | 2.35 | 1.39 | -0.55 | 163 | .586 | .09 |
| We identify words on signs | 1.76 | 1.57 | 2.34 | 1.40 | -2.47 | 157.95 | .015 | .39 |
| I help my child sing/recite the alphabet | 1.80 | 1.41 | 1.90 | 1.28 | -0.49 | 163 | .628 | .07 |
| Literacy Meaning-related Activities |  |  |  |  |  |  |  |  |
| I teach my child the sounds of letters | 2.25 | 1.40 | 2.79 | 1.12 | -2.75 | 161.66 | .007 | .43 |
| I introduce new words and their definitions to my child | 1.73 | 1.38 | 2.65 | 1.20 | -4.55 | 159.77 | <.001 | .71 |
| We make up rhymes in songs | 1.39 | 1.42 | 2.18 | 1.46 | -3.49 | 163 | .001 | .55 |
| I ask questions when we read together | 1.99 | 1.41 | 2.21 | 1.21 | -1.06 | 162 | .290 | .17 |
| We visit the library for children’s books^a^ | 0.55 | 0.98 | 0.34 | 0.75 | 1.55 | 161.99 | .123 | .24 |

*Notes.* Parents responded on a five-point scale to the question: How often do you do the following activities with your child? Response options were 0 - rarely or never, 1- monthly, 2 - weekly, 3 - several days a week, and 4 - most days per week. ^a^This question was not included in any analyses given its low frequency in both groups. *N*s for low-SES = ranged from 93-94, high-SES = 71.

# Appendix B. Detailed Information about Factor Analyses for Parental Activities

**Table B.1**

*Factor Loadings for Parental Academic Expectations (N = 157)*

|  | Factor Loadings | |
| --- | --- | --- |
| Academic Expectations | Literacy | Numeracy |
| Count to 1000 | -.127 | **.918** |
| Multiply | -.166 | **.911** |
| Read printed numbers up to 100 | .169 | **.746** |
| Count to 100 | *.302* | **.560** |
| Solve simple sums | **.559** | .276 |
| Read simple books | ***.536*** | *.339* |
| Print all letters | **.791** | .080 |
| Know some letters | **.799** | -.069 |
| Print name | **.898** | -.198 |
| Know all letters | **.857** | .006 |
| Read some words | **.886** | .004 |

*Notes*. Italicized numbers indicate items that loaded on both factors above .30. The correlation between the numeracy and literacy expectations factors was .668.

**Table B.2**

*Factor Loadings for Parental Numeracy Activities (N = 163)*

|  | Factor Loadings | |
| --- | --- | --- |
| Numeracy Activity | Mapping | Operational |
| Ask about quantities | **.836** | -.020 |
| Recite numbers | **.822** | -.180 |
| Quantities with fingers | **.723** | .079 |
| Teach print numbers | **.580** | .224 |
| Sing counting songs | **.536** | .122 |
| Talk about time | .032 | **.537** |
| Games involve math | .209 | **.592** |
| Weigh, measure | -.031 | **.672** |
| Help simple sums | -.103 | **.767** |
| Math in head | .011 | **.800** |

*Note*. The correlation between the mapping and operational activities factors was .667.

**Table B.3**

*Factor Loadings for Parental Reports of Literacy Activities (N = 162)*

|  | Factor Loadings | |
| --- | --- | --- |
| Literacy Activity | Code-Based | Meaning-Related |
| Help read words | **.917** | -.087 |
| Teach print words | **.873** | -.103 |
| Point letters in words | **.780** | .064 |
| Recognize print letters | **.760** | .082 |
| Recite alphabet | **.563** | .176 |
| Identify words on signs | **.455** | .299 |
| Sounds in letters | *.340* | ***.496*** |
| Teach rhymes | -.032 | **.638** |
| Ask questions when read | .008 | **.746** |
| Learn new words | -.033 | **.806** |

*Notes*. Italicized numbers indicate items that loaded on both factors above .30. The factor correlation between code-based and meaning-related literacy activities was .723.

# Appendix C

**Figure C.1.**

*Path Models for the Full Sample (N = 173)*

| 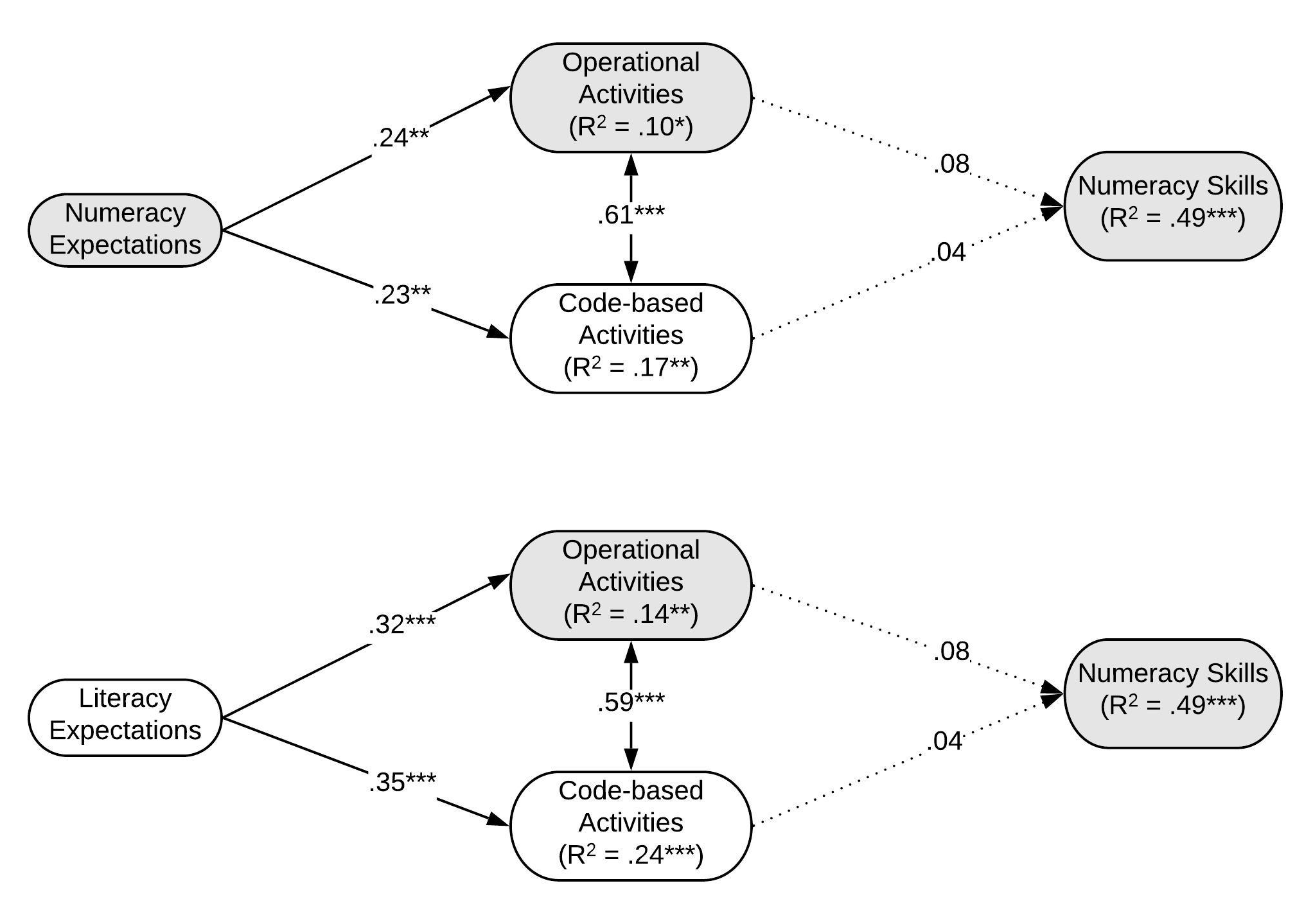 |
| --- |

*Notes.* The top panel is the final path model that involves numeracy expectations. It shows relations among variables for the full sample (*n* = 173) controlling for child’s age: *χ*^2^(1) = 0.87, *p*= .352, SRMR = .01, CFI = 1, RMSEA = 0 (90% CI = [0, .20]). The bottom panel is the final path model that involves literacy expectations. It shows relations among variables for the full sample (*n* = 173) controlling for child’s age: *χ*^2^(1) = 1.53, *p*= .216, SRMR = .02, CFI = 1, RMSEA = .06 (90% CI = [0, .22]). The *R*^2^ values shown in Figure C.1 include variance predicted by the control measure. The numbers on the arrows are the standardized coefficients. Dashed lines present no significant paths. **p*< .05, ***p*< .01, and ****p*< .001.
